# Quantifying social segregation in large-scale networks

**DOI:** 10.1038/s41598-022-10273-1

**Published:** 2022-04-19

**Authors:** Bjørn-Atle Reme, Andreas Kotsadam, Johannes Bjelland, Pål Roe Sundsøy, Jo Thori Lind

**Affiliations:** 1 Centre for Fertility and Health, Norwegian Institute of Public Health, Oslo, Norway; 2 Ragnar Frisch Centre for Economic Research, Oslo, Norway; 3 Telenor Research, Oslo, Norway; 4 Department of Economics, University of Oslo, Oslo, Norway

**Keywords:** Human behaviour, Psychology

## Abstract

We present a measure of social segregation which combines mobile phone data and income register data in Oslo, Norway. In addition to measuring the extent of social segregation, our study shows that social segregation is strong, robust, and that social networks are particularly clustered among the richest. Using location data on the areas where people work, we also examine whether exposure to other social strata weakens measured segregation. Lastly, we extend our analysis to a large South Asian city and show that our main results hold across two widely different societies.

## Introduction

Similar people are more likely to form social ties. This phenomenon, referred to as social homophily, has been documented in several academic disciplines^1–4^. Social homophily is typically based on socially salient characteristics, such as social class, gender, ethnicity, religion, or beliefs and leads to social segregation. Given the importance of social contact for building trust, empathy, and cooperation in a population^5–10^, measuring social segregation is crucial. However, it is difficult as it requires data on the patterns of interpersonal contact in a population. With a few notable exceptions^11–13^, most studies have therefore either proxied social segregation with spatial segregation, or studied social networks in smaller groups^14–18^. While spatial segregation is important, it is insufficient as a proxy for larger scale social segregation. Most importantly, spatial proximity does not imply contact and studies show that closeness without contact may actually increase social divides^14,19–22^.

Technological progress, and particularly the introduction of the smartphone, has allowed for utilizing high granularity mobile phone data. This has also led to a renewed interest in social network patterns^19,23–30^. However, thus far, there have not been studies which combine detailed mobile phone records with income register data to study social segregation, while controlling for spatial proximity. This is the purpose of our study. We make several contributions to the literature on socioeconomic segregation. First, by combining mobile phone data and income register data, we quantify social segregation across income in Oslo, Norway. In particular, we estimate the association between income differences and communication intensity, while controlling for spatial proximity. Second, we examine how daytime exposure to other social strata affects the degree of social segregation. Third, we estimate how social clustering varies across income. Last, by extending the analysis to a large south-east Asian city, we explore the similarities of these patterns across widely different societies.

In contrast to self reported survey data, mobile data reflects actual behavior. In addition, by also including detailed data on spatial proximity in our analyses, this study offers an unprecedented view of actual social segregation. As economic opportunities, such as job market outcomes, are strongly influenced by social networks this segregation may have important consequences for vulnerable groups^1^.

## Data and methods

### Measuring communication

To measure social contact in Oslo we utilize detailed call data records over a 3 month period in 2013 from the market’s largest mobile network operator. This operator has close to 250,000 subscribers in the city area, covering approximately 50% of the population. We define a communication event as any communication in terms of phone call or text message. During the period at hand the subscribers initiated 36 million communication events. While internet-based services for messaging and calling, such as Skype, WhatsApp, Messenger and FaceTime, currently are the primary mode of communication with friends for many, this study is from 2013, a time when the penetration of such services was very limited. For example, a survey for this time period found that among the general population, more than 50% had never used such services, more than 20% used them less than once a month, and approximately 15% used only them 1–3 times per month^31^.

### Measuring socioeconomic status

In order to measure socioeconomic status, we use data on labor earnings from the Norwegian register data, where we have reported earnings for all Norwegian citizens, as well as demographic details.

### Linking communication and socioeconomic status

The study is in accordance with relevant guidelines and regulations. Therefore, due to privacy protection regulations, the call data records and income data were not linkable on the level of the individual. To link these data sources we aggregate the analysis on the level of a mobile base station, of which there are 689 in the sample (see Fig. A.3 in the Supplementary Material for maps showing mobile towers and communication in Oslo). Every cell phone subscriber is associated a *home tower*—the base station most used by the subscriber between 7:00 pm and 7:00 am in this 3 month period. The socioeconomic status of a home tower’s catchment area is defined as the mean income of residents with positive incomes living in the area (see Table A.1; Fig.  A.1 in Supplementary Material for descriptive statistics of the sample).

### Measuring social segregation

The core of our analyses is based on the income and amount of communication between the 474,721 directed pairs of home towers (see Supplementary material Fig. A.2 for the distribution of between tower communication intensities). As our data are on communication between pairs of towers, each with a heterogeneous population of subscribers, and the economic differential between each pair of individuals is continuous, we rely on techniques studying the relationship between aggregate link strengths and aggregate differences in population composition as in^13^.

We assign an average income to each cell tower. We use register data on pre-tax wage for all individuals in Oslo in 2010. We know the basic unit (‘grunnkrets’) of residence of each individual. We can then average over all individuals residing in basic unit *g* to find the average income in that unit, $y_g$. To map incomes to cell towers, we construct cell tower *t*’s coverage by dividing the city into Voronoi polygons^32^. Let $A_{tg}$ denote the area of overlap between basic unit *g* and tower Voronoi polygon *t*. If they don’t overlap, $A_{tg}=0$. Then the estimated income of residents of tower *t*’s catchment area is estimated as

$$\begin{aligned} {\bar{y}}_t=\frac{\sum _g A_{tg}y_g}{\sum _g A_{tg}}. \end{aligned}$$

To summarise our approach, consider the following illustrating example: if an agent identified with tower *t* makes a call from a location associated with tower *u*, then the link is registered as emerging from tower *t*. Moreover, if the receiver lives at location *v*, but receives the call at location *w*, then the link is registered as between location *t* and *v*.

In the analysis of group specific incomes, we use the register data to compute age group and gender specific income averages and we use a similar formula for estimating the average income for this demographic group in tower *t*’s catchment area. One potential challenge with using group averages instead of individual level data is that it can lead to an aggregation bias: the group-level associations misrepresent the individual-level associations^33^. To check the sensitivity of our results with respect to this type of bias, we have undertaken analyses where we impute group averages on income for 12 demographic groups—gender and six age groups. The results indicate that our primary income measure is best at explaining communication patterns (see Supplementary material Table A.5).

### Asian data

We used 1 month of raw call data records from the country’s largest carrier to construct a country-wide call graph. The number of total subscribers were 113 million, 2.7 billion communication links and 10,000 mobile towers. This dataset was further subset to only contain the links for the largest city—covering 18 million subscribers, 111 million social ties and 2974 towers.

For income we used survey data, since no reliable income register data exists. The income categories for a random selection of 76,005 subscribers were obtained through two sequential large-scale market research household surveys. Information about income was directly asked from the respondents, who were requested to place themselves within pre-defined income bins. The survey also contains geo-coordinates of the location of residence of each respondent. Respondents within the household were selected via the Kish grid method among those who were eligible^34^. The correlation between the average income per region based on the survey results and their values published in official statistics were 0.925.

In order to calculate the income distribution at the cell tower level, the dataset was first restricted to contain participants from the largest city. Then income was aggregated on the tower level by assigning every respondent to her closest tower (by bird’s flight).

### Measuring correlations with income differences

To estimate the correlation between income differences and communication intensity, we estimate the regression model

$$\begin{aligned} \text {Events}_{ij}=\alpha + \beta |\ln {\bar{y}}_i-\ln {\bar{y}}_j| + \theta ^{\prime }z_{ij} + \nu _{ij} \end{aligned}$$

Here $\text {Events}_{ij}$ is the number of communication events between towers *i* and *j*, ${\bar{y}}_i$ and ${\bar{y}}_j$ the average income of residents at each tower, $z_{ij}$ a vector of controls and $\nu _{ij}$ the residual. The vector of covariates $z_{ij}$ includes a fourth order polynomial specification of the geographical distance between the cell towers, the logarithm of the income level of the sending and receiving towers, the total tower traffic level of the sending and receiving tower and the expected tower traffic level. The effect of increased income differences on communication intensity is the parameter $\beta$. We also consider fixed effects specifications where the constant term $\alpha$ is replaced by sending and receiving specific intercepts $\alpha _i^S+\alpha _j^R$.

### Measuring the correlation with daytime exposure

An individual residing at a rich nighttime tower and frequenting a poor daytime tower, or vice versa, is said to be exposed to other socioeconomic groups. To measure daytime exposure to other social strata, we first identify each individual’s most used cell tower between 12 am and 2 pm in the weekdays, the *daytime tower*. We then regress the average home tower income of each individual on the average income of the other individuals who are at the same daytime tower. We measure exposure as the individual deviations in absolute terms from this regression line, i.e. the extent to which the income differential between one’s own income and other’s deviates from the expected level. Specifically, we use the log of the absolute value of the residuals ${\hat{e}}_i$ as our measure of exposure. To estimate the modifying role of exposure to other strata, we estimate the modified model

$$\begin{aligned} \text {Events}_{ij}=\alpha + \beta |\ln {\bar{y}}_i-\ln {\bar{y}}_j| + \gamma \ln |{\hat{e}}_i| \times \ln |\ln {\bar{y}}_i-\ln {\bar{y}}_j| + \theta ^{\prime }z_{ij} + \nu _{ij} \end{aligned}$$

yielding a margin effect of an increase in the log income difference of $\beta +\gamma \ln |{\hat{e}}_i|$.

### Measuring clustering

Clustering in different networks is computed as the share of triplets of nodes that are closed (i.e. conditional on A communicating with B and C, B and C also communicate with each other). The data are weighted by communication intensity assigning each triplet a weight proportional to the arithmetic mean of the intensity going out, estimated with the tnet package in R^35^.

## Results

### Income differences and communication intensity

**Figure 1:**
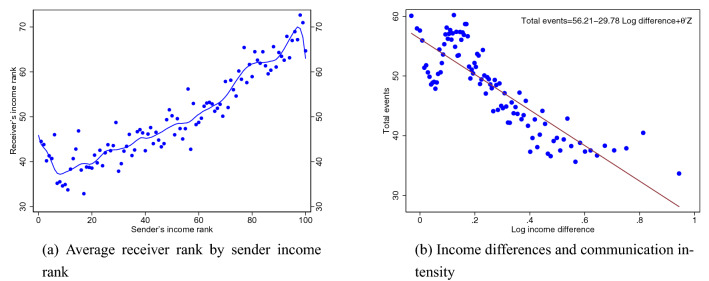
Economic segregation in telecommunication. The figure illustrates economic segregation in telecommunication in Oslo. Panel (**a**) shows the average income rank of receivers by the income percentile of the sender, as well as local linear smoothing of the relationship. Within tower communication is disregarded to exclude within household communication. Panel (**b**) shows a binned scatter plot with the number of communication events between cell phone towers against the absolute value of difference in log income between the two. *Z* includes controls for geographical distance between two cell towers (up to fourth polynomial), the income level of sending and receiving tower, total tower traffic level and expected tower traffic level.

Our main findings are presented in Fig. 1. In Fig. 1a, we plot the average income rank of recipient towers against the income percentile of the senders’ towers. We notice a strong tendency of over-proportional in-group communication, especially among the highest income groups. Except for the extremes of the distribution, the relationship is monotonic.

For instance, the expected income of the communication partner increases by about 15% of average income when moving from the the 1st to the 70th percentile in the income distribution; the effect is comparable when moving from the 70th to the 100th percentile. The variation in sender tower income ranks is higher than the variation in the average receiver rank due to regression toward the mean effects—if communication is to some extent random, senders at the top and bottom percentiles have their average receiver income rank “biased” towards the sample mean. Still, this is a clear indication that the link strength between two nodes in the network is inversely related to their income difference.

Figure 1b quantifies the relationship between communication and income differences, while controlling for spatial distance and a range of other control variables (see Table A.2 in Supplementary Material for the corresponding regression table). A 10% increase in the income difference between two towers is correlated with 2.98 fewer communication events. This is a substantial number, as less than 43% of tower pairs have 3 or more communication events. To assure that the relationship is not driven by communication within family, we exclude communication occurring within the same night time tower from the analysis. However, this communication represents less than a tenth of a percent of events and less than half a percent of the amount of communication, so the effect is negligible. In the Supplementary Material we also report on how the degree of segregation varies across age and gender (Fig. A.4).

### Daytime exposure to other social strata

**Figure 2:**
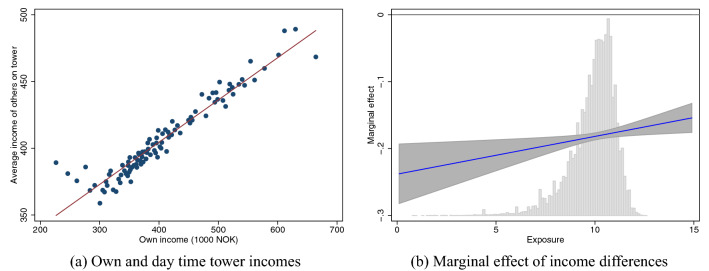
Daytime exposure to other social strata and segregation. Panel (**a**) shows the relationship between own income and average income at the daytime tower. Exposure to other socioeconomic groups is measured as the absolute deviation from this regression line. Panel (**b**) shows the distribution of exposure and the marginal effect of income differences on total communication intensity as a function of exposure.

In this section, we investigate the relationship between exposure to other socioeconomic groups and segregation. We find a strong positive relationship between own income and other’s income ($R^2 = 0.8$), illustrated in Fig. 2a. Hence, spatial income segregation in society is upheld during daytime as well.

Figure 2b reveals a negative correlation of between tower income differentials for all levels of exposure. The positive slope shows that the extent of segregation is decreasing in exposure to other socioeconomic strata (see Table A.3 in Supplementary Material for corresponding regression table). We notice that the total number of events as well as probability of communicating with a tower is less correlated with income differentials for individuals who experience stronger exposure to other income groups during the day as the interaction term is positive. Still, this difference is relatively small in magnitude and there is a negative correlation with income differentials for all groups.

### Clustering within income groups

**Figure 3:**
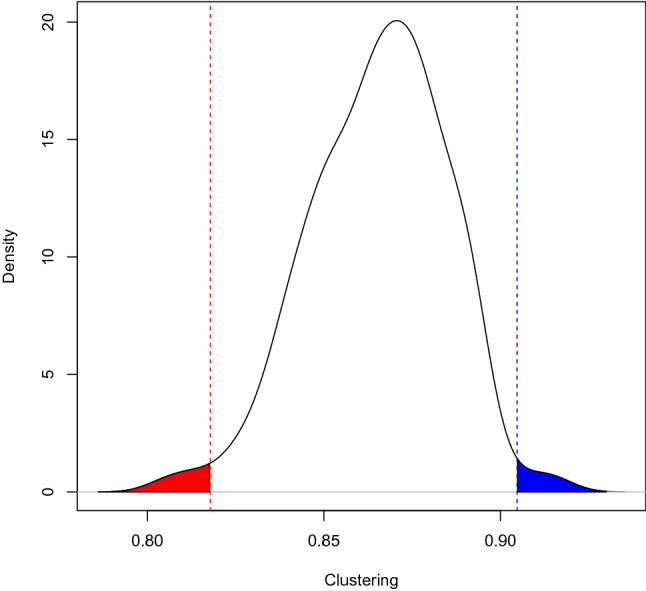
Clustering in networks. The figure shows the distribution of the communication weighted clustering coefficient, in random samples of 100 towers, as well as the clustering coefficient observed in the poorest (red line—to the left) and richest (blue line—to the right) 100 towers.

To study the details of the clustering patterns, we compare the level of clustering among the rich and among the poor. The weighted clustering coefficient among the richest and poorest 100 towers is 0.90 and 0.82, respectively. To put the numbers in perspective, we compute the same coefficient for random draws of 100 towers. In Fig. 3, we show the distribution of the simulated groups as well as the two realized values. Only 1.6% of the simulations are below the value for poorest towers whereas only 1.4% are above the level of the richest towers, indicating very different networks in the two subsamples. In the Supplementary Material we also report the weighted clustering coefficients when using the 25, 50, 100 and 200 poorest and richest towers—the results remain highly stable (Table A.4 in Supplementary Material).

### Correlations in a large South Asian city

**Figure 4:**
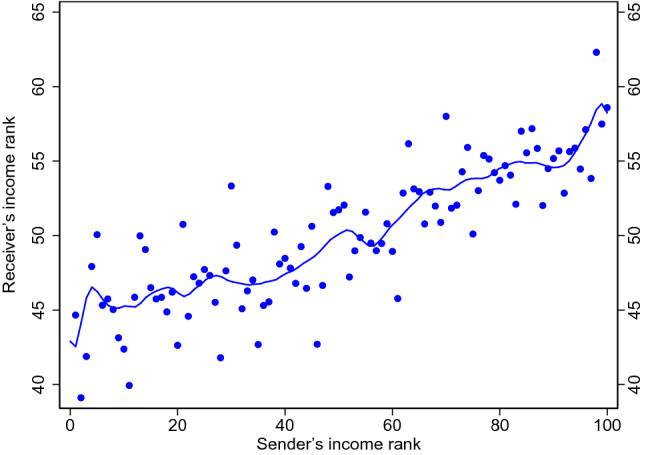
Segregation in the Asian data: average income of sender and receiver. The figure corresponds to Fig. 1a. The figure shows the average income percentile of receivers by the income percentile of the sender as well as local linear smoothing of the relationship. Within tower communication is disregarded.

To verify the external validity of our findings, we undertake the same analysis as in the Norwegian data with the data from a South Asian city. The city is one of the largest in Asia, outside China. Figure 4 replicates Fig. 1a for the Asian data with 111 million links (see Table A.6 in Supplementary Material for corresponding regression table). We find a strikingly similar pattern. Compared to the results for Oslo, it appears that the social segregation among the top 5% is even stronger, and quite extreme compared the rest of the sample. Although the effects of income differences on communication intensity is evident, it is somewhat weaker, possibly because cell phone ownership is less widespread among the poor. This is partially an artifact of the change in the distribution of income between the two countries, but comparing normalized beta coefficients we see that there is a change in effect size as well.

## Discussion

Social contact across groups is important for society since it generates trust and collaboration^8,9^. It is also important at the individual level since networks influence economic opportunities such as finding jobs^1^. Measuring the extent of social contact is difficult as it requires data on the patterns of interpersonal contact in a population. The combination of mobile phone data and income register data carries promise with regards to monitoring the extent and patterns of social segregation.

Our results demonstrate that social segregation is strong in Oslo, in the sense that there is an over-proportional share of communication within income groups. This implies that people from different income groups have less contact with each other. We also examine the extent to which exposure to other socioeconomic groups, measured as neighbourhood during work hours, modifies the social segregation. Social segregation remains strong, even after controlling for daytime exposure to other social strata. Moreover, the degree of social clustering is particularly high among citizens in rich neighborhoods. We also find similar social segregation patterns in a large south-east Asian city.

There are several limitations in this study. First, our data does not allow for any causal interpretations. For instance, in discussing the relationship between exposure to other social strata and the extent to which an individual is socially segregated there may be self-selection whereby more socially integrated individuals choose to work in more socially integrated areas. Second, our measure of daytime exposure to other social strata has an obvious weakness: even though an area is socially diverse, it does not with necessity imply that people interact across socioeconomic groups. Third, whereas income data from Oslo is based on complete registers, the study from a large South East Asian city relies on self reported income. This limits comparability, since self report may be biased and data is likely not missing at random. At the same time, the analysis based on income ranks is less sensitive to this potential weakness.

Future studies should further aim at measuring social segregation over time, something we cannot do with our data due to anonymization. Such a study could perhaps also lead to a better estimate of the causal relationship between exposure and segregation.

## Supplementary Information

Supplementary Information.

## Data Availability

The data that support the findings of this study are available from two different third parties: Statistics Norway and the telecommunications company. Restrictions apply to the availability of these data, which were used under license for the current study, and so are not publicly available. Data are however available from the authors upon reasonable request and with permission of the third parties. The name of the telecommunications company is available from the authors upon request.
