# Supplementary material for: Quantifying social segregation in large-scale networks
Source: Sci Rep. 2022 Apr 19;12:6474. doi: 10.1038/s41598-022-10273-1 (PMC9018874; doi:10.1038/s41598-022-10273-1)
Supplement: Supplementary file 1 — Supplementary Information. [file 41598_2022_10273_MOESM1_ESM.pdf]

Supplementary material

This document is supplementary material for: *Reme, Kotsadam, Bjelland, Sundsøy and Lind: "Quantifying social segregation in large-scale networks" (Scientific Reports)*

List of Figures

A.1 Distribution of communication intensities 4

A.2 The distribution of communication intensity 4

A.3 Maps 5

A.4 Segregation by gender and age in the Oslo data 7

List of Tables

A.1 Descriptive statistics 3

A.2 Quantitative communication intensities – Oslo 6

A.3 Exposure to other groups 6

A.4 Clustering coefficients 7

A.5 Communication intensity and income differences: Effect of own income 8

A.6 Segregation in the Asian data 9

The expected amount of communication

Some towers are more heavily used than others, among others due to their location and the number of nearby towers. Hence there is more communication going in and out of some towers than others, and hence some tower links are more intense simply because of the importance of the two towers. To separate this effect from effects due to segregation, we construct a measure of expected communication between two towers, based on the amount of outgoing communication from the sender tower and income communication ate the receiving tower.

The expected amount of communication is calculated as follows: Let T be the total number of communication events in the whole network, T_A be the number of events originating at tower A, and T_B the number of events directed at tower B. Then the probability that an events originates from tower A is $\frac{T_A}{T}$ and the probability that an event is directed at B is $\frac{T_B}{T}$. If the two events are independent, the probability that a particular event goes from A to B is hence $\frac{T_A T_B}{T^2}$ and the expected number of events between the two towers $\frac{T_A T_B}{T}$.

Table A.1: Descriptive statistics

A. Individual characteristics

	N	Mean	Median	Std.dev.	Min	Max
Income at home tower	243064	403324.2	381882.5	90662.68	208885.6	694299.9
Age	171825	46.54782	46	16.45585	8	104
Woman	171442	.4854761	0	.4997905	0	1
Total events	243064	580.3309	397	691.2333	1	47968
Voice events	243064	209.0512	114	333.8863	0	47968
SMS events	243064	363.1634	207	537.9726	0	30258
MMS events	243064	8.116233	2	25.57441	0	6728

B. Dyad characteristics

	N	Mean	Median	Std.dev.	Min	Max
Total events	467856	77.37444	0	860.1191	0	145953
Difference in log income	467856	.274112	.2342548	.2049387	0	1.201117
Geographic distance	467856	6.383163	5.543356	4.287963	0	38.49146

Note. The Table reports descriptive statistics for the data used in this study.

Panel A. shows data at the level of the individual subscriber. Note that data on age and gender is missing from a part of the sample.

Panel B. shows data at the level of the tower dyad.

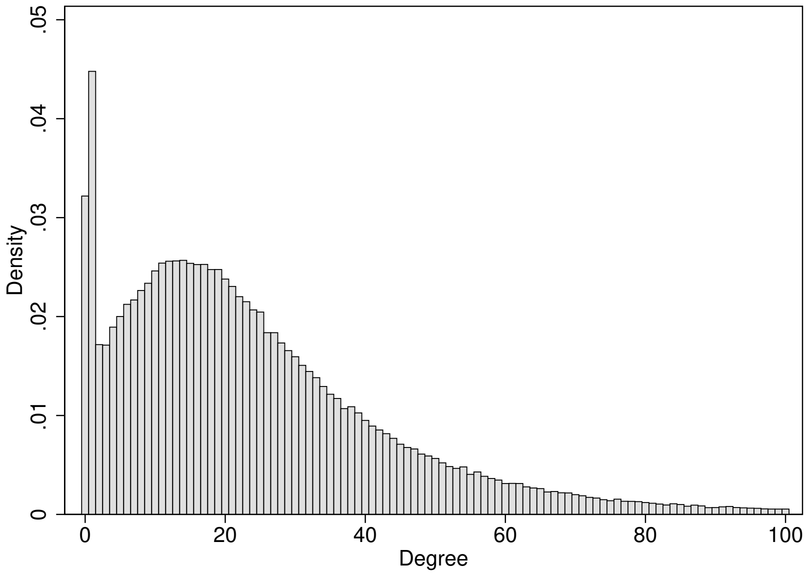

Figure A.1: Distribution of communication intensities

Note. The figure shows the distribution of outward communication intensities (events initiated during the 3 month period) from all individuals in the sample. The data are capped at 100 events.

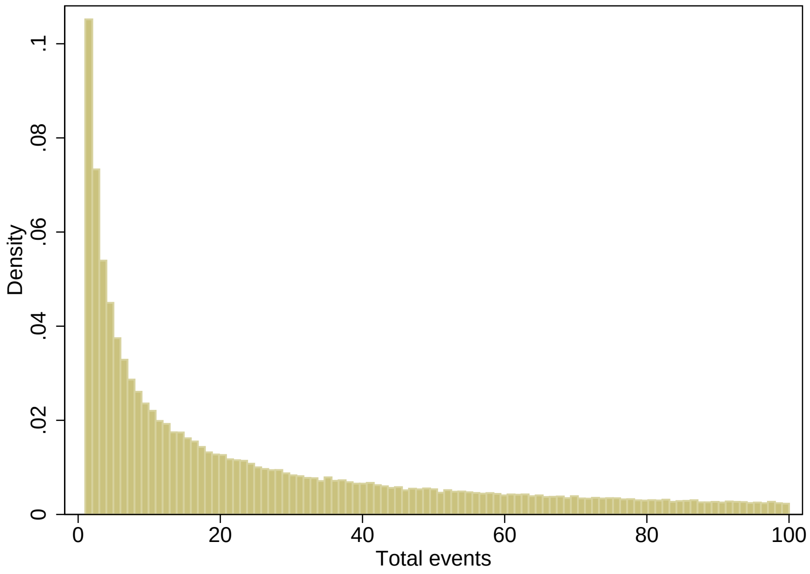

Figure A.2: The distribution of communication intensity

Note. The histogram shows the density of positive communication intensities and is capped at 100 events.

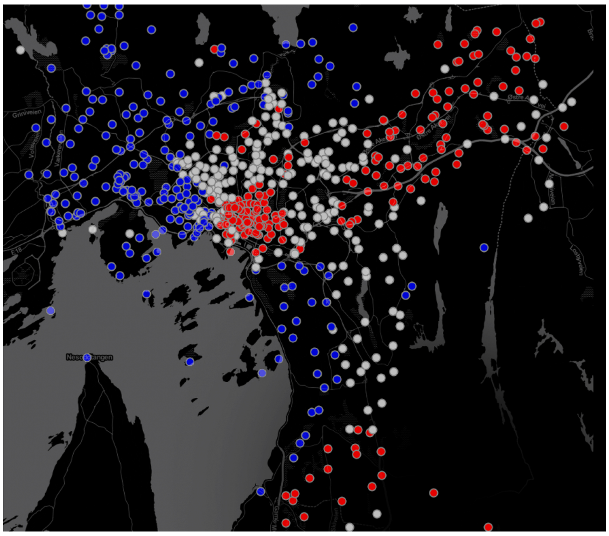

(a) Mobile towers with income levels

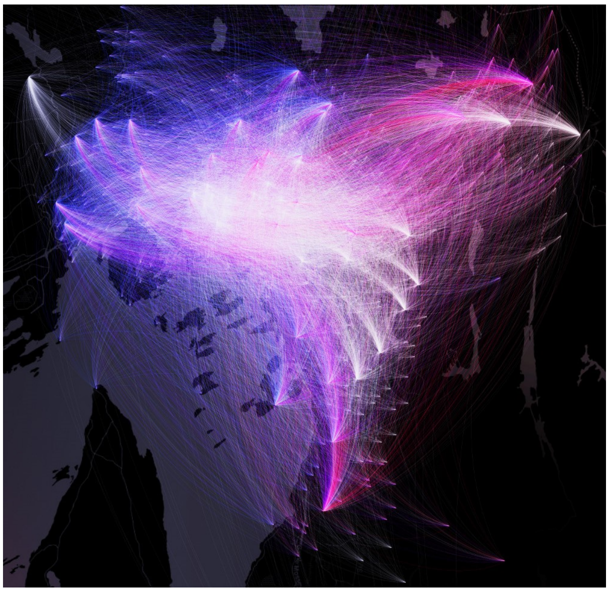

(b) Expected communication intensity

Figure A.3: Maps

Note. The Figure illustrates additional geographic aspects of social segregation in telecommunication in Oslo. Panel (a) shows the mobile towers in Oslo. Ranked by the incomes of residents surrounding the tower, the richest 3rd is shown in blue, the middle 3rd in white and the poorest 3rd in red. Panel (b) shows the 50 strongest *expected* links from each tower overlaid a map of Oslo. Communication from the richest 3rd of towers is shown in blue, the middle 3rd in gray, and the poorest 3rd in red. The figure was generated by the authors using the open-source software Gephi v.0.9.1 (https://gephi.org/) combined with a base map from OpenStreetMap (https://www.openstreetmap.org/).

Table A.2: Quantitative communication intensities – Oslo

	(1)	(2)	(3)	(4)	(5)
Difference in log mean income	-29.78***	-47.28***	-24.85***	-0.707***	-0.713***
	(-39.54)	(-29.09)	(-5.37)	(-42.21)	(-42.09)
Obs	465067	467172	465067	465067	467172
R2	0.430	0.266	0.450		
Alpha				5.423	5.522
Trim at 1500	Yes	No	Yes	Yes	No
Estimator	OLS	OLS	FE	Neg. bin.	Neg. bin.

Note. The table shows how communication intensity can be explained by differences in income. The dependent variable is the number of communication events between the two cell phone towers. The regressions control for geographical distance between the cell towers (up to fourth polynomial), the income level of sending and receiving tower, total tower traffic level and expected tower traffic level.
Reported coefficients are coefficients with t-values in parenthesis, and *, **, and *** denotes significant at the 10 percent, 5 percent, and 1 percent levels.
Columns (1) and (2) report results from OLS regressions whereas Column (3) reports results from a regression where we include tower fixed effects for the sending and receiving tower. In this specification, standard errors are clustered two-ways on sending and receiving tower. Columns (4) and (5) report results from negative binomial regressions.

Table A.3: Exposure to other groups

	(1) Total events	(2) Extensive margin	(3) Intensive margin
Difference in log mean income	-0.238***	-0.0121***	-1.091
	(-10.39)	(-29.55)	(-0.52)
Exposure	0.00574***	0.000299***	0.242***
	(7.57)	(22.08)	(4.33)
Exposure * Inc. difference	0.00564**	0.000478***	-0.421**
	(2.51)	(11.90)	(-2.06)
Obs	126566843	126567413	1630912
R2	0.003	0.022	0.010

Note. The table shows how communication intensity can be explained by differences in income. In the column ("Total events"), the dependent variable is the number of communication events between the two cell phone towers, in column 2 ("Extensive margin") a dummy variable for communication occurring, and in column 3 ("Intensive margin") the number of events conditional on communication occurring.
Reported coefficients are coefficients with t-values in parenthesis, and *, **, and *** denotes significant at the 10percent, 5percent, and 1percent levels. A range of control variables are included, such as geographical distance between two cell towers (up to fourth polynomial), the income level of sending and receiving tower, total tower traffic level and expected tower traffic level.

Figure A.4: Segregation by gender and age in the Oslo data

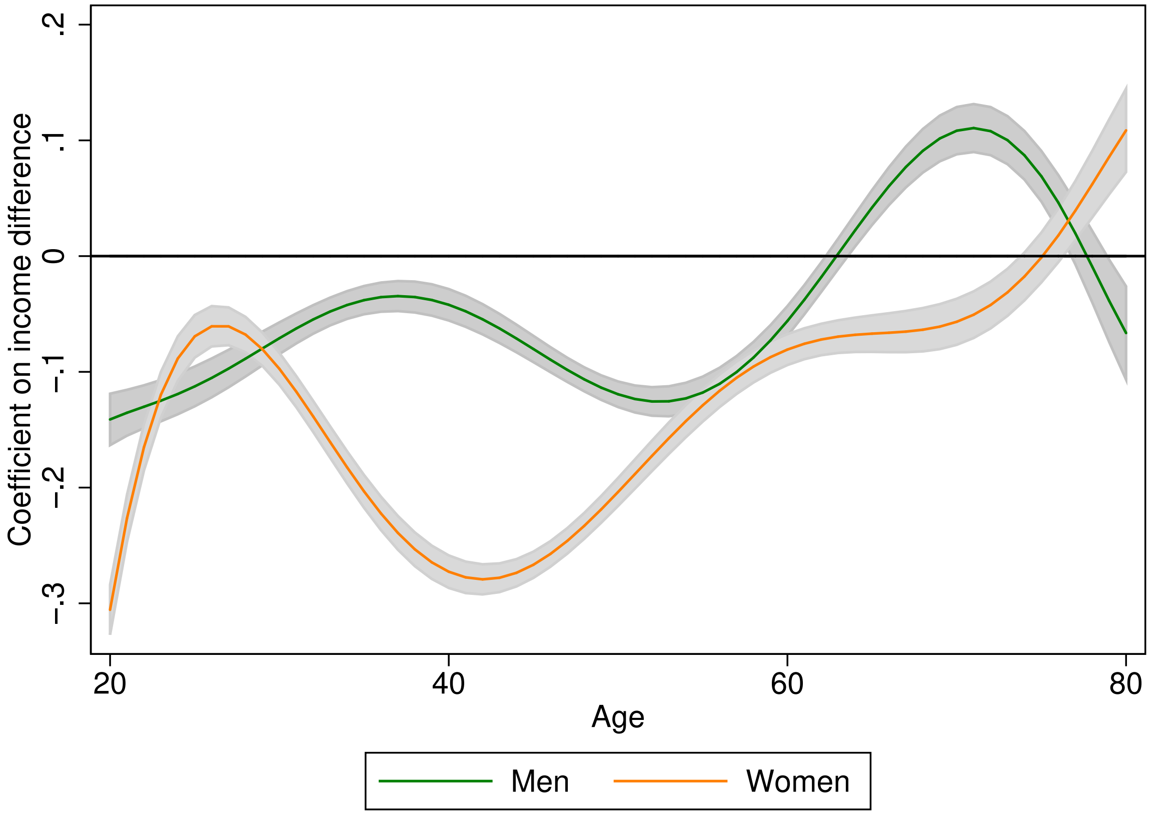

Note. The figures shows the relationship between social segregation and age. Communication from an individual to a cell phone tower is regressed in the log absolute income difference between the sender and receiver tower. The regression coefficient is modelled as a gender specific 7 dimensional polynomial in sender age. A lower coefficient indicates more segregation.
Grey areas are 95 percent confidence bands. Estimation is based on a 7 dimensional polynomial in age.

Table A.4: Clustering coefficients

# towers	Bottom	Top
25	0.825	0.915
50	0.837	0.91
100	0.818	0.905
200	0.844	0.91

Note. The Figure shows the weighted clustering coefficient, in random samples of 25, 50, 100 and 200 poorest ("Bottom") and richest ("Top") towers.

Table A.5: Communication intensity and income differences: Effect of own income

	(1)	(2)	(3)	(4)	(5)
Mean income					
Log mean income, station A	-1.967		21.41***	26.83***	11.22***
	(-1.22)		(8.44)	(10.54)	(4.33)
Log mean income, station B	-2.808		19.95***	23.27***	16.88***
	(-1.74)		(7.84)	(9.13)	(6.63)
Difference in log mean income	-63.12***		-64.50***	-132.9***	-44.34***
	(-32.41)		(-22.45)	(-13.30)	(-11.16)
Age × difference				1.417***	
				(6.83)	
Woman × difference					-48.20***
					(-8.42)
Own income					
Log own income, sender		-23.44***	-25.84***	-23.99***	-17.63***
		(-21.68)	(-21.59)	(-20.01)	(-13.67)
Log own income, receiver		-18.32***	-20.66***	-20.11***	-19.03***
		(-16.83)	(-17.07)	(-16.60)	(-15.62)
Difference in log own income		-7.490***	-1.611	10.26*	2.820
		(-6.00)	(-1.23)	(2.53)	(1.54)
Age × difference				-0.144	
				(-1.81)	
Woman × difference					-2.599
					(-1.01)
N	1681732	906621	906621	906621	906621
r2	0.0460	0.0494	0.0501	0.0511	0.0507

Note. The table shows how communication intensity can be explained by differences in income measured as the tower average and the estimated individual income. The latter is estimated by imputing group averages at the basic unit level based on gender and six age groups for a total of 12 demographic groups.
Reported coefficients are coefficients with t-values in parenthesis, and *, **, and *** denotes significant at the 10%, 5%, and 1% levels. A range of control variables are included, such as geographical distance between two cell towers (up to fourth polynomial), the income level of sending and receiving tower, total tower traffic level and expected tower traffic level.

Table A.6: Segregation in the Asian data

	(1)<br>Total events	(2)<br>Extensive margin	(3)<br>Intensive margin
Difference in log mean income	-2.066***	-0.00797***	-2.753***
	(-47.76)	(-28.03)	(-39.42)
Obs	8825652	8841702	5317734
R2	0.144	0.200	0.137

Note. The Table corresponds to Table A.2. Column (1) measures total communication from tower A to tower B, Column (2) employs a dummy for the presence of communication, and Column (3) only considers dyads with communication. Income is normalized my mean individual income.

Reported coefficients are coefficients with t-values in parenthesis, and *, **, and *** denotes significant at the 10percent, 5percent, and 1percent levels. A range of control variables are included, such as geographical distance between two cell towers (up to fourth polynomial), the income level of sending and receiving tower, total tower traffic level and expected tower traffic level.
